# 

**Published:** 1909-08-21

**Authors:** 


### BOOKS RECEIVED.

W. B. Saunders Co.

"Dietetics for Nurses"—2nd edition, by Julius Friedenwald, M.D.

"Primary Studies for Nurses," by Charlotte A. Aikens.

"Obstetrics for Nurses," by Joseph B. DeLee, M.D.

### Charitable Bequest.

Miss Mary Ann Hutchinson, of Avenue Parade, Accrington, who died on June 28, left estate valued at £30,562 gross, with net personalty £29,967. The testatrix left £100 each to the Wesleyan Missionary Society, the Victoria Cottage Hospital, and the Union Street, Accrington, Chapel Fund.

### GRADUATES' MEDICAL SCHOOLS.

DIARY FOR THE WEEK, AUGUST 23 to 28.

**THE POST-GRADUATE COLLEGE, West London Hospital, Hammersmith, W.**

**At 10 a.m.**
**August 23 and 26,** Surgical Registrar, **Demonstration.**
**August 27,** Medical Registrar, **Demonstration,**

**At 12 noon.**
**August 23,** Dr. Bernstein, **Pathological Demonstration.**

**At 12.15 p.m.**
**August 24,** Dr. Pritchard, **Practical Medicine.**
**August 25,** Dr. Grainger Stewart, **Practical Medicine.**

**At 5 p.m.**
**August 24,** Dr. Grainger Stewart, **Syphilitic Diseases of Nervous System.**
**August 25,** Mr. Bishop Harman, **Squint.**

### APPOINTMENTS.

Dr. T. S. Higgins has been appointed assistant medical officer of health for Birmingham.

Dr. W. O. Arnold has been appointed Deputy Medical Officer of Health to the Gateshead Corporation.

Dr. Dudley Buxton has been appointed Lecturer in Anæsthetics at the Royal Dental Hospital, London.

Dr. E. H. S. Nash, of Derby, has been appointed Medical Officer of Health and School Medical Officer of Wimbledon.

Dr. Sinclair, of Middlesbrough, has been appointed School Medical Officer to the Gloucester Education Committee.

Dr. William Savage has been appointed Medical Officer of Health and Chief Medical Inspector of Schools for the County of Somerset.

Dr. Raines (assistant medical officer of health for Hull) has been appointed deputy-medical officer to the Hull and Goole Port Sanitary Authority.

Dr. Patrick T. McArdle, Assistant Master to the National Maternity Hospital, Holles Street, Dublin, has been appointed Gynæcologist to the hospital.

Dr. J. R. Lambert has been appointed medical officer of the newly-formed medical relief district of Farsley, in the North Bierley Union; and Dr. Norman Hughes has been elected medical officer of the newly-formed relief district of Calverley in the same Union.

Mr. Sydney Scott, M.S., F.R.C.S., has been appointed Surgeon for Diseases of the Throat and Ear at the National Hospital for the Paralysed and Epileptic, Queen Square. Mr. Scott holds the position of Chief Assistant in the Aural Department at St. Bartholomew's Hospital, and is Aural Surgeon to the Evelina Hospital for Children.

**THE HOSPITAL** August 21, 1909.

*Name* ..........................................

*Address* ..........................................

..........................................

This Coupon must accompany manuscript or contributions intended for The Hospital.

